# BTK抑制剂、维奈克拉联合利妥昔单抗治疗慢性淋巴细胞白血病/小淋巴细胞淋巴瘤的疗效及安全性

**DOI:** 10.3760/cma.j.issn.0253-2727.2023.03.012

**Published:** 2023-03

**Authors:** 睿 姜, 罗梦佳 戴, 业钦 沙, 奕 夏, 祎 缪, 姝超 秦, 微 吴, 婧妍 邱, 红岭 秘, 莉 王, 磊 范, 卫 徐, 建勇 李, 华渊 朱

**Affiliations:** 1 南京医科大学第一附属医院，江苏省人民医院血液科，南京 210029 Department of Hematology, the First Affiliated Hospital of Nanjing Medical University, Jiangsu Province Hospital, Nanjing 210029, China; 2 江苏省人民医院浦口分院血液科，浦口慢淋中心，南京 211800 Pukou CLL Center, Pukou Division of Jiangsu Province Hospital, Nanjing 211800, China

慢性淋巴细胞白血病（CLL）/小淋巴细胞淋巴瘤（SLL）在我国发病率逐年升高，随着布鲁顿酪氨酸激酶（BTK）抑制剂（BTKi）、抗凋亡蛋白Bcl-2抑制剂维奈克拉（Ven）等的临床应用，CLL治疗模式逐渐从以FCR（氟达拉滨+环磷酰胺+利妥昔单抗）、BR（苯达莫司汀+利妥昔单抗）、苯丁酸氮芥联合CD20单抗为主的化学免疫治疗（CIT）向小分子靶向药物的持续治疗模式转变。持续性单药治疗模式下有限的缓解深度（特别是BTKi单药）、经济负担、患者依从性及耐药相关靶点基因突变等因素促使新药治疗时代的有限疗程疗法成为未来治疗的探索方向[1]。本研究分析报道我中心8例满足治疗指征的CLL/SLL一线接受BTKi、Ven联合利妥昔单抗的疗效及安全性。

## 病例与方法

1. 病例：2020年3月至2022年3月期间，8例达到治疗指征的CLL/SLL患者在南京医科大学第一附属医院浦口慢淋中心一线接受ZVR（泽布替尼+Ven+利妥昔单抗）、IVR（伊布替尼+Ven+利妥昔单抗）或OVR（奥布替尼+Ven+利妥昔单抗）方案治疗，其中CLL 7例，SLL 1例。分别有4例、2例和2例患者接受ZVR、IVR和OVR方案。诊断标准参照《中国慢性淋巴细胞白血病/小淋巴细胞淋巴瘤的诊断与治疗指南（2018年版）》[2]，所有患者均进行骨髓或外周血流式细胞术免疫分型、骨髓病理形态学及免疫组化检查。CLL按照Rai和Binet分期进行临床分期，SLL按照Ann Arbor分期进行临床分期。采用慢性淋巴细胞白血病国际预后指数（CLL-IPI）评分系统进行预后评估[3]。

2. 治疗方案：BTKi具体为泽布替尼160 mg每日2次、伊布替尼420 mg每日1次或奥布替尼150 mg每日1次，自第1周期第1天开始口服。Ven自第3周期第1天开始，20 mg每日1次，按照50、100、200 mg每日1次爬坡至最终剂量400 mg每日1次。利妥昔单抗375 mg·m^−2^·d^−1^第1天，500 mg·m^−2^·d^−1^，第8、15天（第1周期），500 mg·m^−2^·d^−1^第1天（第2～6周期），28 d为1个周期。在BTKi、Ven联合治疗满9个月时行外周血和骨髓微小残留病（MRD）检测，外周血和骨髓均获得阴性者在联合治疗满12个月时再次确认为阴性则停药随访。

3. 疗效及安全性评价：CLL疗效评估参照《中国慢性淋巴细胞白血病/小淋巴细胞淋巴瘤的诊断与治疗指南（2018年版）》[2]，SLL疗效评估参照2014版Lugano[4]，流式细胞术检测MRD，评估总缓解率（ORR）、完全缓解（CR）率、部分缓解（PR）率、MRD及不良反应事件（AE）。AE根据CTCAE 5.0进行分级。MRD阴性根据iwCLL 2018版指南[5]定义为每10 000个白细胞中CLL细胞<1个；高MRD水平为MRD水平≥10^−2^；低MRD水平为MRD水平≥10^−4^而<10^−2^；MRD阴性为MRD水平<10^−4^。肿瘤溶解综合征（TLS）风险评估主要根据基线放射学评估淋巴结大小和淋巴细胞绝对计数[6]。

4. 随访：采用电话随访。末次随访时间2022年3月8日，中位随访时间18.4（9.5～24.0）个月。

5. 统计学处理：计量资料用“中位数（范围）”表示，计数资料用例数表示。

## 结果

1. 基本特征：8例患者中位年龄为54.5（40～67）岁，其中男7例，女1例。CLL Rai分期Ⅱ期3例，Ⅲ期1例，Ⅳ期3例；Binet分期B期3例，C期4例。1例SLL为Ann Arbor分期Ⅳ期B组。IGHV无突变6例，复杂核型（CK）3例（例4、6、8），TP53异常4例（例2、4、5、8），其中2例（例2和例4）合并del（17p）和TP53突变。CLL-IPI评分低危、中危、高危及极高危分别为1例、1例、2例和3例。8例基线TLS风险评估高风险1例，中风险6例，低风险1例，经前两个月导入期后再次评估TLS风险，其中中风险3例，低风险5例（表1）。

**表1:** 8例初治慢性淋巴细胞白血病（CLL）/小淋巴细胞淋巴瘤（SLL）患者的临床特征

例号	性别	年龄（岁）	诊断	Rai分期	Binet分期	CLL-IPI	IGHV			FISH异常	核型	基因突变
							突变状态	使用片段	符合率（％）			
1	男	40	CLL	Ⅳ	C	1	有突变	3-48*04	96.60	无异常	46, XY[15]	SMARCA4
2	女	47	CLL	Ⅲ	C	7	无突变	2-70*01	100	del(17p)、del(13q14)	45, XX, der(13;17)(q10;q10)[5]/46, XX[5]	TP53、SF3B1、SETD2
3	男	67	CLL	Ⅳ	C	6	无突变	1-69*01	100	无异常	46, XY[20]	NOTCH1、FAT1
4	男	56	CLL	Ⅱ	B	7	无突变	1-69*01	100	del(17p)	45, XY, der(4;17)(q10;q10)[5]/42, idem,−Y, t(2;8)(q21;q24),−4,−20[1]/44, idem,−Y, add(15)(p11)[1]/43, idem, −Y, der(5;14)(q10; q10), add(6)(p25)[1]/46, XY[2]	TP53
5	男	57	CLL	Ⅱ	B	5	无突变	1-69*01	100	del(11q)、del(13q14)	46, XY, del(11q14)[10]	TP53
6	男	53	SLL	–	–	–	无突变	1-69*01	100	IGH缺失	45, XY, der(6)t(6;14)(p21; q11), del(9)(q13),−14[3]/46, idem, +3[1]/46, XY[6]	NOTCH2
7	男	47	CLL	Ⅱ	B	3	有突变	3-7*01	92.20	del(13q14)	46, XY, del(13)(q14)[6]/46, XY[4]	SETD2
8	男	62	CLL	Ⅳ	C	9	无突变	4-34*01	100	del(13q14)	46, XY, del(1)(q31), t(1;3)(q31;q29), der(2), t(10;21)(p10;q10), del(13)(q22)[10]	TP53、NOTCH1

**注** –：不适用

2. 疗效：1例SLL患者外周血无肿瘤细胞，未纳入MRD随访。整组患者中，BTKi和Ven的中位联合治疗时间为15.6（7.5～21.6）个月，最佳ORR为100％（8/8），最佳CR率为75％（6/8），最佳外周血MRD阴性率为85.7％（6/7），其中5例（71.4％）同时达骨髓MRD阴性。BTKi联合Ven、利妥昔单抗治疗满3个月时，ORR为100％，5例患者接受骨髓评估，2例达到CR，3例达到PR。7例评估外周血MRD，4例达低MRD水平，1例同时达外周血及骨髓MRD阴性。BTKi联合Ven、利妥昔单抗治疗满9个月时，4例检测外周血者均达MRD阴性。至末次随访，6例患者BTKi联合Ven、利妥昔单抗治疗已达到1年，3例（例2、例4、例5）达CR/CR伴骨髓不完全恢复（CRi）及外周血、骨髓MRD阴性，1例（例1）因脾大评估为PR但外周血和骨髓同时达MRD阴性，1例（例3）为CR但外周血和骨髓MRD分别为0.02％和0.04％，1例（例8）因疫情未按计划完成所有利妥昔单抗治疗（仅在第1个周期治疗2次），在BTKi联合Ven满6个月时达外周血和骨髓MRD阴性，1年时随访缺失，但在联合治疗满18个月时外周血和骨髓MRD复阳（均为0.06％），加用奥妥珠单抗。1例SLL（例6）在BTKi联合Ven治疗满6个月时达到CR。8例CLL/SLL的治疗流程图见图1。

**图1:**
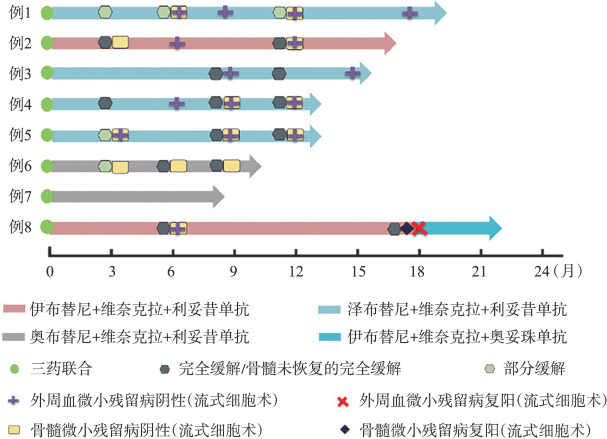
8例初治慢性淋巴细胞白血病/小淋巴细胞淋巴瘤的治疗流程 例6为SLL患者，未纳入外周血微小残留病随访

3. 安全性评价：最常见的3～4级血液学不良反应为中性粒细胞减少（3例），非血液学不良反应为纤维蛋白原减少（1例）和肌酸激酶升高（1例）。最常见的1～2级血液学不良反应为血小板减少（4例）和中性粒细胞减少（2例），非血液学不良反应为出血（3例）、斑丘疹（2例）。Ven爬坡过程中均未发生TLS。6例患者在治疗过程中因AE调整剂量或停药：3例（例3、例6和例7）分别因咽部出血2级AE、皮下血肿2级AE、斑丘疹2级AE短暂停用泽布替尼6 d和奥布替尼15 d、2 d；1例（例4）因服用伊布替尼后出现阵发性房性心动过速2级AE、肌酸激酶升高3级AE和高磷酸血症2级AE，伊布替尼减量至280 mg后仍不能耐受，改为泽布替尼未出现不耐受；1例（例8）因持续中性粒细胞减少4级AE将Ven减量至300 mg每日1次维持治疗并间断应用粒细胞集落刺激因子支持；1例（例5）因持续中性粒细胞减少4级AE将Ven减量至200 mg每日1次维持治疗，后因纤维蛋白原降低3级AE及血便2级AE而停用泽布替尼16 d。

## 讨论

小分子靶向药物BTKi、Bcl-2抑制剂Ven的出现使CLL/SLL的治疗模式从传统的CIT时代进入了无化疗时代。既往研究表明FCR方案使体能状态良好且免疫球蛋白重链可变区（IGHV）有突变的年轻患者生存获益[7]，但合并TP53异常、IGHV无突变等不良生物学因素的患者无法从传统CIT中长期获益。BTKi及Ven已被报道在复发难治CLL和初治CLL中均有显著疗效且可部分克服不良生物学因素[8]–[11]，但持续性单药（特别是BTKi单药）治疗模式下的有限缓解深度、经济负担、患者依从性及耐药进展等问题日益突出[12]–[14]，因此固定周期或以MRD为指导的BTKi及Ven联合或不联合CD20单抗治疗已成为小分子靶向治疗时代有限疗程方案的未来探索趋势。本研究初次报道了中国初治CLL/SLL患者应用BTKi、Ven联合利妥昔单抗的疗效及安全性。

Rogers等[15]探索固定14个周期IVO方案（伊布替尼+Ven+奥妥珠单抗）治疗25例初治CLL患者的疗效及安全性，ORR为84％，14例患者同时获得外周血及骨髓MRD阴性，其中7例疗效评估达CR，中位随访时间为41.4个月，3年PFS率及OS率均为95％。MRD指导下的泽布替尼、Ven联合奥妥珠单抗（BOVen）在39例初治CLL患者的临床试验中，中位随访25.8个月，最佳CR率为57％，89％的患者在中位联合治疗8个月后达到预设MRD标准而停药并中位随访15.8个月，94％的患者持续外周血MRD阴性，2例MRD转阳[16]。CLL2-GIVe研究探索了IVO方案治疗41例合并TP53异常的高危CLL患者的疗效及安全性，中位随访26.6个月，第15个周期CR率、外周血及骨髓MRD阴性率分别为58.5％、78％和65.9％，24个月PFS率及OS率均为95.1％，中位PFS时间为33.5个月，中位OS时间未达到。21例在第14或15个周期达到预设停药条件而随访，其中4例在第27～36个周期疾病进展[17]。上述方案均提示，在具备一线治疗指征的CLL患者中，固定周期或以MRD为指导的BTKi、Ven及CD20单抗三药联合方案所获得的CR率与CIT时代相比差异无统计学意义，但均能取得较高的外周血及骨髓MRD阴性率，且在合并IGHV-UM及TP53异常患者中差异无统计学意义；即使不同方案下药物联合应用时长不一，但上述三药联合方案在CLL有限疗程方案的探索中具有深远前景，也揭示MRD作为PFS替代观察指标的可能性。

本中心首次报道在中国CLL/SLL患者中探索一线应用BTKi、Ven联合利妥昔单抗治疗的初步结果，本队列中CLL-IPI高危/极高危患者占71.4％，IGHV-UM、TP53异常者分别占75％及37.5％，即使使用的BTKi有所不同。中位随访18.4个月，所有患者最佳ORR及CR率分别为100％及75％，外周血MRD阴性率为85.7％，71.4％的患者同时达到外周血和骨髓MRD阴性。值得关注的是，所有患者在联合治疗满3个月时早期检测外周血MRD均已达到低MRD水平，其中1例（例5）患者在ZVR方案联合治疗满3个月时即获得外周血和骨髓MRD阴性，在联合治疗满9个月及12个月时连续确认达CR伴外周血及骨髓MRD阴性并进入停药随访期。在目前联合治疗达1年的6例患者中，3例达到CR，1例达到PR，伴外周血和骨髓MRD阴性。除上述ZVR方案治疗的患者外，3例（例1、例2、例4）患者等待再次确认疗效及MRD水平，其中包括2例（例2和例4）TP53异常者。余1例TP53异常者（例8）联合治疗满6个月时达到外周血和骨髓MRD阴性，但联合治疗满18个月时MRD复阳而选择加用奥妥珠单抗。与国外的BTKi、Ven联合奥妥珠单抗的队列数据相比，本队列中的高危及极高危、IGHV-UM及TP53异常患者比例均不低于既往研究，尽管因药物可及性而选择BTKi、Ven联合利妥昔单抗治疗，但早期仍能较快地清除MRD，最佳外周血及骨髓MRD阴性率与同等随访时长的AVG方案队列研究接近，联合治疗1年后在包括TP53异常的大部分患者中可获得CR/PR伴外周血及骨髓MRD阴性并尝试停药。未来在联合用药的时间、固定周期或是MRD指导下的停药时机选择、MRD检测深度、再次启动治疗的时机及此类治疗下克隆演变等方面仍需进一步探索。

在安全性方面，既往国外报道，BTKi、Ven联合奥妥珠单抗治疗下3～4级中性粒细胞减少的发生率为18％～56％[16]–[17]，与本研究3～4级中性粒细胞减少的发生率相似。另外，本研究中有5例患者出现BTKi相关AE，较既往单药研究的发生率高[8],[18]，均有不同程度的调整剂量，但未发生因AE永久停药，其中有1例患者（例3）合并持续性房颤，选用靶点选择性更高的泽布替尼，且起始剂量为80 mg每日1次，在后续随访过程中未再次出现房颤，另外1例患者出现纤维蛋白原降低3级AE，既往大多数BTKi、Ven注册研究中未将纤维蛋白原指标列入随访指标，故未见相应AE报道，此例患者经短暂停用BTKi后未再次出现该AE，考虑可能与BTKi药物相关，纤维蛋白原降低的发生率及严重程度尚待在真实世界中进一步观察。本研究8例初治患者应用BTKi、Ven联合利妥昔单抗方案安全性可控，耐受性好，但仍需要长期监测。

本研究首次报道了在包括TP53异常等高危因素的中国CLL/SLL患者中应用BTKi、Ven联合利妥昔单抗的疗效及安全性，该类方案可使患者获得早期深度缓解且有望使部分患者停药，持续缓解期及远期生存获益有待长期随访以明确。
